# Medical Rats

**Published:** 1880-07

**Authors:** 


MEDICAL RATS.—Men who habitually violate the "fee-tables" of their respective communities, when opportunity is offered for advancing their interests, have been designated by some of our exchanges as "Rats." It would be interesting to know how many of these *rodents* we have in our midst?

